# Supplementary material for: Causal interplay between lactose intolerance and gut microbiota: a combined bidirectional Mendelian randomization and in vivo validation study
Source: Front Nutr. 2026 Jun 1;13:1803337. doi: 10.3389/fnut.2026.1803337 (PMC13265576; doi:10.3389/fnut.2026.1803337)
Supplement: Supplementary file 1 [file Data_Sheet_1.zip › supplementary materials/Forward/leave one out cross validation/ebi-a-GCST90027548.finngen_R12_E4_LACTONAS.pdf]

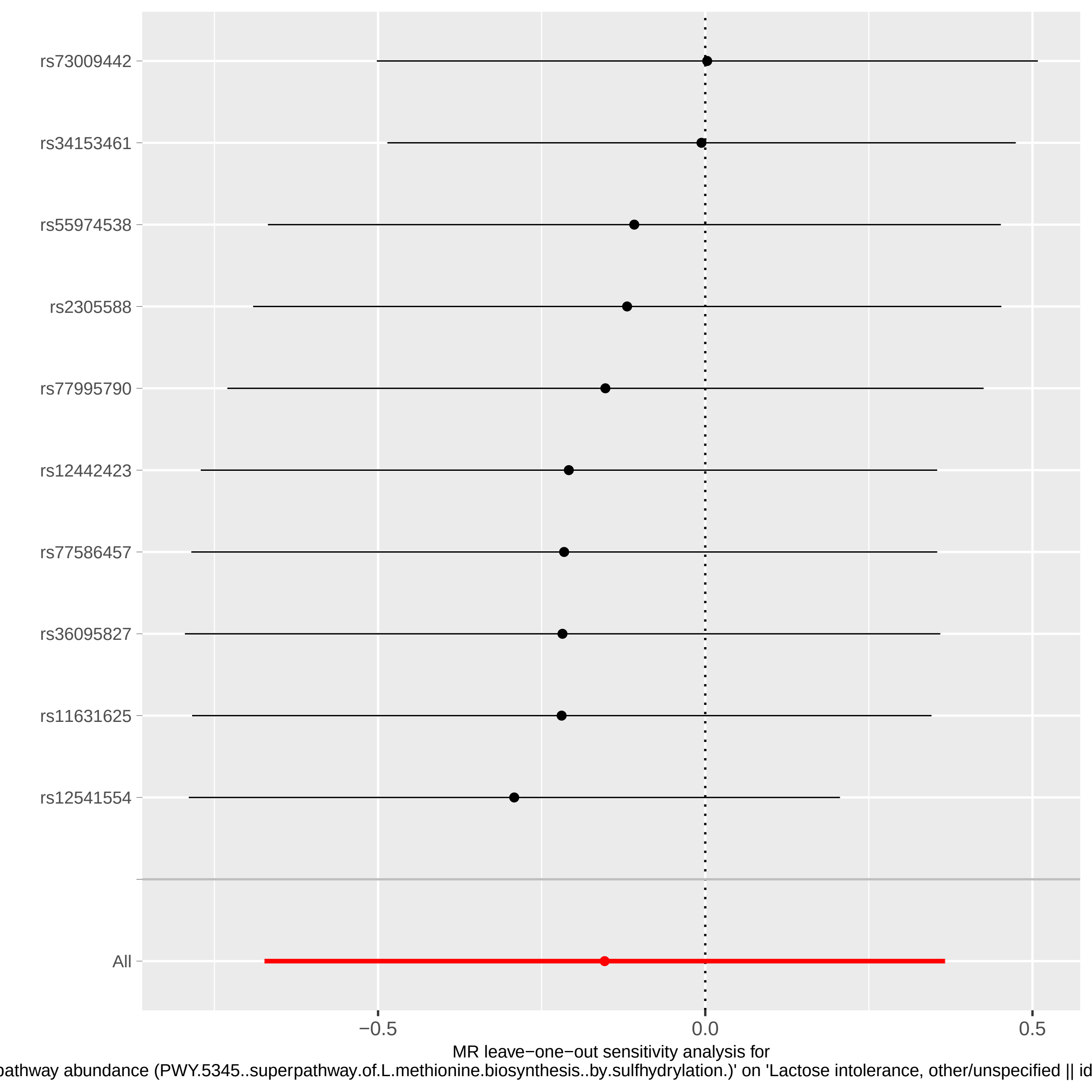

rs73009442
rs34153461
rs55974538
rs2305588
rs77995790
rs12442423
rs77586457
rs36095827
rs11631625
rs12541554
All
−0.5
0.0
0.5
MR leave−one−out sensitivity analysis for
pathway abundance (PWY.5345..superpathway.of.L.methionine.biosynthesis..by.sulfhydrylation.)' on 'Lactose intolerance, other/unspecified || id
